# Association between total cholesterol and lumbar bone density in Chinese: a study of physical examination data from 2018 to 2023

**DOI:** 10.1186/s12944-023-01946-5

**Published:** 2023-10-21

**Authors:** Yongbing Sun, Xin Qi, Xinbei Lin, Yang Zhou, Xue Lv, Jing Zhou, Zhonglin Li, Xiaoling Wu, Zhi Zou, Yongli Li, Hao Li

**Affiliations:** 1 Department of Medical Imaging, People’s Hospital of Zhengzhou University, #7 Wei Wu Road, Zhengzhou, 450003 Henan People’s Republic of China; 2 Department of Medical Imaging, Henan Provincial People’s Hospital, Xinxiang Medical College, Zhengzhou, Henan China; 3 Department of Health Management, Chronic Health Management Laboratory, Henan Provincial People’s Hospital, Zhengzhou, Henan China; 4 Henan Provincial People’s Hospital, Zhengzhou, Henan China; 5 Department of Nuclear Medicine, Henan Provincial People’s Hospital, Zhengzhou, Henan China; 6 Department of Health Management, Fuwai Central China Cardiovascular Hospital, #1 Fuwai Avenue, Zhengzhou, Henan China

**Keywords:** Osteoporosis, Bone mineral density, Total cholesterol, Chinese adults

## Abstract

**Background:**

The impact of total cholesterol (TC) on lumbar bone mineral density (BMD) is a topic of interest. However, empirical evidence on this association from demographic surveys conducted in China is lacking. Therefore, this study aimed to examine the relationship between serum TC and lumbar BMD in a sample of 20,544 Chinese adults between the ages of 20 and 80 years over a period of 5 years, from February 2018 to February 2023. Thus, we investigated the effect of serum TC level on lumbar BMD and its relationship with bone reduction in a Chinese adult population.

**Methods:**

This cross-sectional study used data obtained from the Department of Health Management at Henan Provincial People’s Hospital between February 2018 and February 2023. The aim of this study was to examine the correlation between serum TC and lumbar BMD in individuals of different sexes. The research methodology encompassed population description, analysis of stratification, single-factor and multiple-equation regression analyses, smooth curve fitting, and analysis of threshold and saturation effects. The R and EmpowerStats software packages were used for statistical analysis.

**Results:**

After adjusting for confounding variables, a multiple linear regression model revealed a significant correlation between TC and lumbar BMD in men. In subgroup analysis, serum TC was found to have a positive association with lumbar BMD in men, specifically those aged 45 years or older, with a body mass index (BMI) ranging from 24 to 28 kg/m^2^. A U-shaped correlation arose between serum TC and lumbar BMD was detected in women of different ages and BMI, the inflection point was 4.27 mmol/L for women aged ≥ 45 years and 4.35 mmol/L for women with a BMI of ≥ 28 kg/m^2^.

**Conclusion:**

In this study, Chinese adults aged 20–80 years displayed different effects of serum TC on lumbar BMD in sex-specific populations. Therefore, monitoring BMI and serum TC levels in women of different ages could prevent osteoporosis and osteopenia.

**Trial registration:**

The research protocol was approved by the Ethics Committee of Beijing Jishuitan Hospital, in accordance with the Declaration of Helsinki guidelines (No. 2015-12-02). These data are part of the China Health Quantitative CT Big Data Research team, which has been registered at clinicaltrials.gov (code: NCT03699228).

**Supplementary Information:**

The online version contains supplementary material available at 10.1186/s12944-023-01946-5.

## Introduction

Total cholesterol (TC) is a vital lipid constituent of the human body and has a critical impact on bone cell metabolism [1]. Recent studies have proposed the concept of cholesterol toxicity, which influences organs through activation of inflammation, mitochondrial dysfunction, and endoplasmic reticulum stress [2]. A preclinical study using quantitative computed tomography in mice found that a high-cholesterol diet can lead to low bone content [3]. Research based on data from the US National Health and Nutrition Examination Survey (NHANES) database has shown that TC is inversely associated with lumbar bone mineral density (BMD) in older non-cancer individuals aged ≥ 60 years [4]. Another study discovered a correlation between low TC levels and reduced total BMD in young adults aged 20–29 years; in individuals aged 40–49 years and those with borderline diabetes, a non-linear curve associating TC with total BMD was identified, with an inflection point at 4.65 mmol/L and 6.7 mmol/L, respectively [5]. A study of 1,116 Chinese women showed a non-linear association between TC and lumbar BMD among postmenopausal women, and a negative correlation between them when TC < 5.86 mmol/L [6]. All the above-mentioned studies suggest that TC may be related to BMD, but little data have been obtained from China. Therefore, it is necessary to explore the correlation between serum TC and lumbar BMD in Chinese individuals from a holistic perspective.

Osteoporosis is a chronic condition marked by a heightened prevalence of generalized BMD loss, affecting approximately 200 million people worldwide [7] and approximately 90 million people in China suffer from this disease [8]. Osteoporosis is commonly classified into primary and secondary types, and BMD reduction can be used as a diagnostic index [9]. Researchers typically evaluate the progression of osteoporosis using lumbar BMD measurements. Given the high prevalence and harmful effects of osteoporosis, investigating the effect of TC on BMD is critical. Studies have indicated that women are generally at a greater risk of osteoporosis than men, and that most men tend to have larger, stronger bones than women and experience less bone loss throughout their lives [10]. In recent years, the relationship between TC and BMD has been explored and is a concern worldwide; however, the results remain inconsistent because of geographical and sample size limitations, which indicates that race has a great impact on the prevalence of osteoporosis. Therefore, the homogenous study population and reasonable sample size in this study can assist in clarifying the effects of TC on BMD in Chinese adults. This information may provide guidance for preventing and treating BMD loss in Chinese adults. To our knowledge, this is the first study to investigate the association between lumbar BMD and TC in a Chinese population of different sexes using a large dataset of medical examinations and performing a subgroup analysis.

This study gathered data from individuals who underwent physical examinations over 5 consecutive years at Henan Provincial People’s Hospital in China to explore the linear or non-linear correlation between TC and BMD in different sexes. Covariates, such as blood pressure and BMD-related biochemical test results, were filtered to improve sample quality and corresponding analysis.

## Materials and methods

### Study participants and inclusion criteria

The study’s analysis relied on physical examination data from the Health Management Department of Henan Provincial People’s Hospital collected between February 2018 and February 2023. These data were part of the China Health Quantitative CT Big Data Research project team, which has been registered at clinicaltrials.gov (code: NCT03699228). The inclusion criteria were: (1) age between 20 and 80 years; (2) complete information on lumbar BMD and blood biochemical examination; and (3) complete body mass index (BMI) and general demographic information. The exclusion criteria were as follows: (1) history of various cancers, (2) previous or current thyroid disease and other endocrine diseases, (3) previous or current liver or kidney disease, and (4) past or present use of osteoporosis-preventing drugs and lipid-regulating agents. The trained personnel obtained fundamental data through in-person surveys, including age, sex, nationality, medical history, and medication history of the participants.

**Fig. 1:**
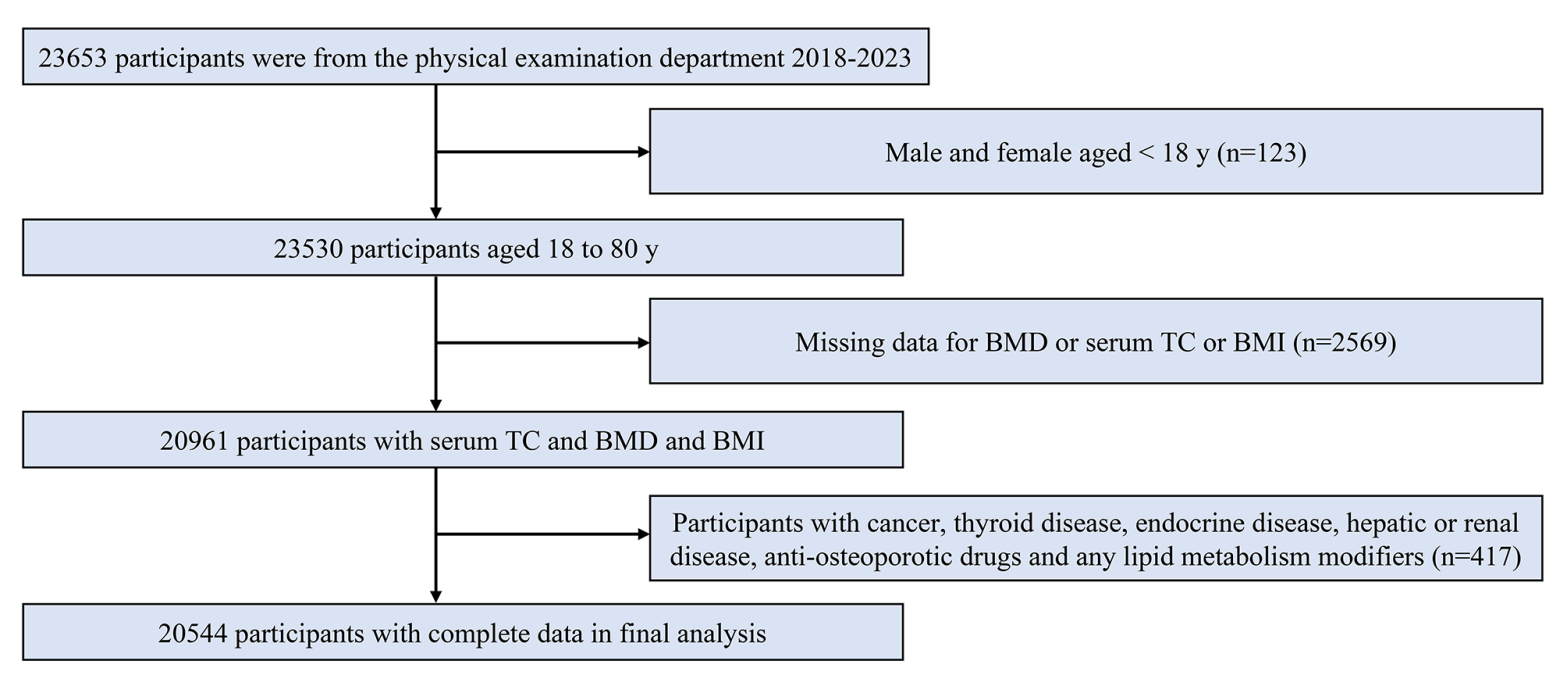
Flowchart of participants selection

A total of 23,653 participants were included, 123 of whom were aged < 20 years and were thus excluded from the study. In addition, 2,569 participants had incomplete lumbar BMD, serum TC, or BMI data, and 417 had medical histories that did not meet the inclusion criteria. Finally, 20,544 participants were included in the study. The flowchart of subject screening is shown in Fig. 1.

### Research methods

All researchers were provided uniform training before the investigation to ensure the precision and impartiality of the data. A standardized questionnaire was used to gather fundamental data from the participants. including the patient’s prior and present medical history, such as a history of cancer, liver disease, kidney disease, thyroid, and other endocrine disorders, and the use of anti-osteoporosis drugs and lipid metabolism regulators. The data were summarized, checked, verified, and proofread after the completion of the questionnaire.

The participants’ height, weight, and blood pressure were measured in the morning after fasting for over 12 h with light clothing and no shoes. Each subject was measured twice and averaged to reduce errors. BMI = weight divided by height^2^ (kg/m^2^).

### Laboratory measurements

Fasting blood samples were collected to measure TC and other laboratory markers, including high-density lipoprotein cholesterol (HDL-C), triglyceride (TG), low-density lipoprotein cholesterol (LDL-C), total protein (TP), total bilirubin (TB), blood phosphorus, blood potassium, blood calcium, alkaline phosphatase (ALP), alanine aminotransferase (ALT), aspartate aminotransferase (AST), fasting blood glucose (FBG), and glycated hemoglobin (GH). An Olympus® AU 5400 automated biochemical analyzer (Olympus Corporation, Japan, Shizuoka) was used to assess the blood glucose and lipid levels. Conventional laboratory techniques were used to assess the remaining indicators.

### BMD measurement

Low-dose chest CT (LDCT) scanning was part of the participants’ routine health examination, with each participant undergoing the same LDCT procedure. Quantitative Computed Tomography (QCT) volume BMD (vBMD) was measured using Mindways QCT Pro (Mindways Software, Inc., Austin, TX, USA), while all CT scans were performed at 120 kVp. The LDCT images were sent to the QCT station for analysis. Lumbar (L1-L2) trabecular vBMD (mg/cm^3^) was determined using asynchronous BMD calibration and QCT Pro analysis (Mindways Software Inc., Austin, USA). All analyses were performed by experienced and trained radiologists using QCT software. This procedure necessitates post-imaging analysis of conventional LDCT images. Therefore, no additional radiation dose was required. A published study validates that these criteria are appropriate for Chinese people [11].

Quality control was upheld consistently during the research period via routine calibration and cross-calibration between systems utilizing a European spinal prosthesis (ESP-145). The results of quality assurance indicated that the mean variation in ESP vBMD detected at each center was < 5 mg/cm^3^.

### Variables

In this investigation, TC and BMD were used as independent and dependent variables, respectively. The following categorical variables were included as covariates: Nationality and marital status. The following dimensions were included as covariates in this analysis: age, BMI, systolic blood pressure (SBP), diastolic blood pressure (DBP), HDL-C, TG, LDL-C, TP, TB, blood phosphorus, blood potassium, blood calcium, ALP, ALT, AST, FBG, and GH.

### Statistical analysis

All data were analyzed using EmpowerStats (X&Y Solutions, Inc., Boston, MA, USA) and the statistical package R (The R Foundation, version 3.6.3). In the final evaluation, the baseline characteristics of all subjects were described by means or medians and quartiles (continuous variables) or proportions (categorical variables). The chi-square test and variance estimation were employed to deal with significant discrepancies in this dataset. The association between serum TC level and lumbar BMD was examined using a multiple linear regression model. The study employed a multivariate linear regression model to perform a subgroup analysis of the linear relationship between serum TC and lumbar BMD among diverse sex groups, categorized by BMI and age. Smooth curve fitting and the generalized additivity model were used to define the non-linear correlation between serum TC level and lumbar BMD. For non-linear situations in the model, objective calculations were conducted to determine the point of inflection in the correlation between serum TC and lumbar BMD, and a two-stage linear regression model was then established on either side of this point. Statistical significance was achieved at a two-tailed *P* < 0.05.

The serum TC frequency distribution graph was created using Origin software (OriginLab, USA, version2022b).

## Result

### Participant baseline characteristics

**Fig. 2:**
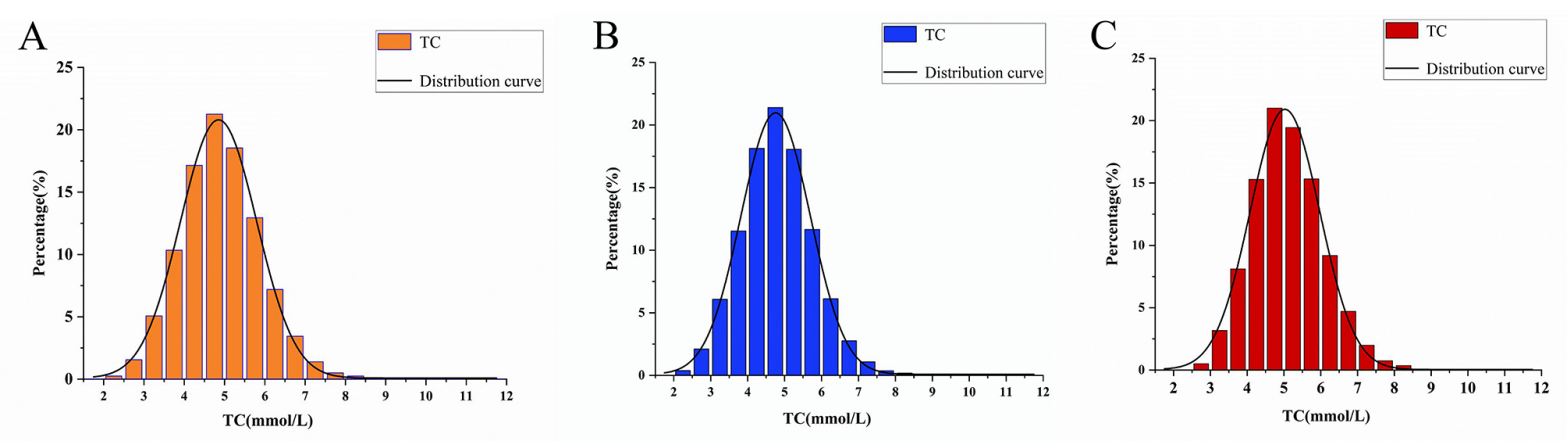
Distribution histogram of TC. **(A)** all participants; **(B)** all males; **(C)** all females; TC, total cholesterol

A total of 20,544 individuals aged 20 to 80 years, comprising 13,389 males and 7,155 females, were recruited for the study. The characteristics of male and female participants were determined by serum TC (Q1: 1.88–4.14, Q2: 4.15–4.77, Q3: 4.78–5.41, Q4: 5.42–12.83; Q1: 1.89–4.44, Q2: 4.45–5.04, Q3: 5.05–5.7, Q4: 5.71–13.77) divided into quartiles. As presented in Table 1, notable variations in baseline characteristics were noted between quartiles of serum TC, aside from nationality, serum phosphorus, and serum potassium in men and nationality and serum potassium in women. Compared with the other subgroups, it is likely that the men with the highest fourth serum TC levels were younger, Han, and married, with higher SBP, DBP, lipid indices (HDL-C, TG, LDL-C, and TP), ALP, ALT, AST, FBG, and lumbar BMD; participants with the highest quartile of serum TC in women were probably older, Han, and married, with higher SBP, DBP, lipid indices (HDL-C, TG, LDL-C, and TP), TP, serum calcium, serum potassium, ALP, ALT, AST, GH, FBG, and lumbar BMD. The serum TC distribution of all participants, males, and females, is shown in Fig. 2.

**Table 1:** Characteristics of the study population

Male (n = 13,389)					
TC (mmol/L)	Q1(1.88–4.14)	Q2(4.15–4.77)	Q3(4.78–5.41)	Q4(5.42–12.83)	*P* value
Age (year)	57.87 ± 13.32	53.31 ± 12.47	52.97 ± 11.40	52.01 ± 10.91	<0.001^***^
Nationality (%)					0.143
Han nationality	99.52	99.07	99.41	99.29	
non-Han nationality	0.48	0.93	0.59	0.71	
Marital status (%)					<0.001^***^
Married	97.35	97.00	97.57	94.80	
Not married	2.65	3.00	2.43	5.20	
DBP (mmHg)	76.25 ± 11.21	78.25 ± 11.62	79.45 ± 11.90	80.76 ± 11.95	<0.001^***^
SBP (mmHg)	130.82 ± 17.96	130.30 ± 18.20	130.64 ± 18.22	132.13 ± 18.27	<0.001^***^
BMI (kg/m^2^), (%)					0.001^**^
<24	33.57	32.79	31.36	29.74	
≥ 24, <28	50.06	48.56	49.18	51.89	
≥ 28	16.37	18.65	19.46	18.37	
HDL-C (mmol/L)	1.17 ± 0.24	1.24 ± 0.25	1.29 ± 0.27	1.33 ± 0.29	<0.001^***^
TG (mmol/L)	1.52 ± 0.84	1.76 ± 1.02	1.99 ± 1.24	2.61 ± 2.21	<0.001^***^
LDL-C (mmol/L)	1.93 ± 0.39	2.62 ± 0.32	3.08 ± 0.36	3.74 ± 0.63	<0.001^***^
TP (g/L)	70.58 ± 3.99	70.97 ± 3.94	71.56 ± 3.84	72.64 ± 4.27	<0.001^***^
TB (µmol/L)	13.00(10.10,16.80)	12.70(9.80,15.90)	12.40(9.80,15.90)	12.20(9.50,15.60)	<0.001^***^
Serum phosphorus (mmol/L)	1.04 ± 0.03	1.04 ± 0.03	1.04 ± 0.03	1.04 ± 0.03	0.693
Serum calcium (mmol/L)	2.34 ± 0.02	2.34 ± 0.03	2.34 ± 0.02	2.34 ± 0.03	<0.001^***^
Serum potassium (mmol/L)	4.23 ± 0.11	4.23 ± 0.08	4.23 ± 0.09	4.23 ± 0.09	0.635
ALP (U/L)	67.21 ± 17.96	68.2357 ± 17.93	67.99 ± 17.41	69.17 ± 18.51	<0.001^***^
ALT (U/L)	25.48(15.90,29.20)	25.14(15.90, 29.10)	26.01(15.90,30.50)	28.78(17.10,32.85)	<0.001^***^
AST (U/L)	22.95(17.50,25.40)	22.47(17.50, 24.90)	22.87(17.70,25.00)	24.17(18.25,26.40)	<0.001^***^
GH (%)	6.03 ± 0.85	5.89 ± 0.80	5.92 ± 0.76	6.00 ± 0.92	<0.001^***^
FBG (mmol/L)	5.58 ± 1.35	5.37 ± 1.31	5.46 ± 1.38	5.65 ± 1.75	<0.001^***^
BMD (mg/cm^3^)	117.04 ± 34.89	123.26 ± 34.93	123.69 ± 33.39	125.38 ± 32.43	<0.001^***^
Female (n = 7155)					
TC (mmol/L)	Q1(1.89–4.44)	Q2(4.45–5.04)	Q3(5.05–5.70)	Q4(5.71–13.77)	*P* value
Age (year)	52.86 ± 13.19	51.26 ± 10.93	53.87 ± 9.75	56.43 ± 9.24	<0.001^***^
Nationality, (%)					0.163
Han nationality	98.81	98.61	97.87	98.01	
non-Han nationality	1.19	1.39	2.13	1.98	
Marital status (%)					<0.001^***^
Married	96.44	96.65	98.21	98.99	
Not married	3.56	3.35	1.79	1.01	
DBP (mmHg)	70.39 ± 11.55	71.63 ± 11.34	72.53 ± 11.42	73.88 ± 11.62	<0.001^***^
SBP (mmHg)	124.28 ± 20.90	123.82 ± 20.20	125.98 ± 20.22	129.03 ± 20.89	<0.001^***^
BMI (kg/m^2^), (%)					0.002^**^
<24	60.51	58.42	56.48	56.97	
≥ 24, <28	30.70	33.24	31.19	33.65	
≥ 28	8.79	8.34	9.33	9.38	
HDL-C (mmol/L)	1.36 ± 0.26	1.46 ± 0.29	1.52 ± 0.31	1.59 ± 0.33	<0.001^***^
TG (mmol/L)	1.27 ± 0.68	1.40 ± 0.85	1.55 ± 0.83	1.77 ± 1.03	<0.001^***^
LDL-C (mmol/L)	2.06 ± 0.38	2.66 ± 0.33	3.13 ± 0.36	3.87 ± 0.59	<0.001^***^
TP (g/L)	71.14 ± 4.06	71.50 ± 3.88	72.10 ± 3.79	72.91 ± 3.91	<0.001^***^
TB (µmol/L)	15.60(11.70,21.40)	15.6(12.00, 21.80)	16.00(12.60,21.50)	16.90(13.20,22.90)	<0.001^***^
Serum phosphorus (mmol/L)	1.17 ± 0.03	1.15 ± 0.05	1.17 ± 0.02	1.16 ± 0.04	0.002^**^
Serum calcium (mmol/L)	2.35 ± 0.06	2.33 ± 0.02	2.35 ± 0.01	2.36 ± 0.03	<0.001^***^
Serum potassium (mmol/L)	4.09 ± 0.07	4.13 ± 0.05	4.19 ± 0.06	4.20 ± 0.08	0.486
ALP (U/L)	65.34 ± 23.49	67.45 ± 22.75	70.33 ± 21.60	72.14 ± 20.44	<0.001^***^
ALT(U/L)	21.02(15.90,23.20)	20.84(16.10,23.10)	21.48(16.90,23.50)	22.54(17.80,24.60)	<0.001^***^
AST(U/L)	19.85(12.60,21.90)	20.44(13.20, 22.00)	22.36(13.65,23.50)	24.28(14.50,25.90)	<0.001^***^
GH (%)	5.85 ± 0.70	5.78 ± 0.57	5.83 ± 0.60	5.90 ± 0.74	<0.001^***^
FBG (mmol/L)	5.17 ± 1.10	5.04 ± 0.93	5.14 ± 1.05	5.29 ± 1.32	<0.001^***^
BMD (mg/cm^3^)	130.18 ± 46.20	132.49 ± 42.46	124.94 ± 42.60	115.98 ± 39.06	<0.001^***^

DBP, diastolic blood pressure; SBP, systolic blood pressure; BMI, body mass index; TC, total cholesterol; HDL-C, high-density lipoprotein cholesterol; TG, triglycerides; LDL-C, low-density lipoprotein cholesterol; TP, total protein; TB, total bilirubin; ALP, alkaline phosphatase; ALT, alanine aminotransferase; AST, aspartate aminotransferase; GH, glycosylated hemoglobin; FBG, fasting blood glucose; BMD, bone mineral density; n, number of subjects; %, weighted percentage

*P < 0.05, **P < 0.01, ***P < 0.001

### Univariate analysis

Univariate analysis demonstrated a negative correlation between lumbar BMD and age, marital status, SBP, HDL-C, ALP, FBG, serum potassium, and GH levels in the male cohort. In contrast, BMI, DBP, LDL-C, TG, TP, ALT, and AST levels demonstrated a favorable correlation with elevated lumbar BMD. In the female cohort, age, marital status, BMI, DBP, SBP, LDL-C, TG, TB, ALT, AST, ALP, FBG, and GH levels were negatively correlated with lumbar BMD. HDL-C level was associated with high lumbar BMD. Table 2.

**Table 2:** The results of univariate analysis

	Statistics	Effect size (*β*)	*P* value
Male (n = 13,389)			
Age (year)	54.03 ± 12.27	-1.56 (-1.60, -1.52)	< 0.001 ^***^
Nationality, (%)			
Han nationality	99.32	Reference	
non-Han nationality	0.68%	3.98 (-3.05, 11.00)	0.267
Marital status (%)			
Not married	3.32	Reference	
Married	96.68	-26.21 (-29.40, -23.02)	< 0.001 ^***^
BMI (kg/m^2^)	25.44 ± 3.02	0.85 (0.66, 1.04)	< 0.001 ^***^
DBP (mmHg)	78.69 ± 11.79	0.05 (0.01, 0.10)	0.028 ^*^
SBP (mmHg)	130.98 ± 18.17	-0.30 (-0.33, -0.27)	< 0.001 ^***^
LDL-C (mmol/L)	2.85 ± 0.79	3.96 (3.23, 4.68)	< 0.001 ^***^
TG (mmol/L)	1.97 ± 1.49	1.53 (1.14, 1.92)	< 0.001 ^***^
HDL-C (mmol/L)	1.26 ± 0.27	-6.89 (-9.03, -4.74)	< 0.001 ^***^
TP (g/L)	71.43 ± 4.09	0.57 (0.43, 0.71)	< 0.001 ^***^
TB (µmol/L)	12.60 (9.80, 16.20)	0.02 (-0.08, 0.13)	0.627
ALT (U/L)	21.60 (16.10, 30.40)	0.15 (0.13, 0.18)	< 0.001 ^***^
AST (U/L)	21.00 (17.70, 25.30)	0.06 (0.02, 0.10)	0.004 ^**^
ALP (U/L)	68.18 ± 17.97	-0.15 (-0.18, -0.12)	< 0.001 ^***^
FBG (mmol/L)	5.52 ± 1.46	-2.26 (-2.65, -1.87)	< 0.001 ^***^
Serum potassium (mmol/L)	4.23 ± 0.09	-1.17 (-7.51, 5.17)	0.718
Serum calcium (mmol/L)	2.34 ± 0.03	14.10 (-8.90, 37.09)	0.230
Serum phosphorus (mmol/L)	1.04 ± 0.03	3.03 (-16.52, 22.57)	0.761
GH (%)	5.96 ± 0.84	-5.42 (-6.11, -4.74)	< 0.001 ^***^
Female (n = 7,155)			
Age	53.62 ± 11.03	-2.85 (-2.90, -2.78)	< 0.001 ^***^
Nationality, (%)			
Han nationality	98.35	Reference	
non-Han nationality	1.65	4.15 (-3.61, 11.91)	0.295
Marital status (%)			
Not married	2.39	Reference	
Married	97.61	-42.78 (-49.18, -36.38)	< 0.001 ^***^
BMI (kg/m^2^)	23.65 ± 3.11	-2.07 (-2.38, -1.75)	< 0.001 ^***^
DBP (mmHg)	72.12 ± 11.53	-0.57 (-0.65, -0.48)	< 0.001 ^***^
SBP (mmHg)	125.78 ± 20.61	-0.78 (-0.82, -0.73)	< 0.001 ^***^
LDL-C (mmol/L)	2.93 ± 0.79	-5.44 (-6.69, -4.20)	< 0.001 ^***^
TG (mmol/L)	1.50 ± 0.88	-7.89 (-9.01, -6.78)	< 0.001 ^***^
HDL-C (mmol/L)	1.49 ± 0.31	4.08 (0.91, 7.25)	0.011 ^*^
TP (g/L)	71.90 ± 3.97	0.04 (-0.29, 0.21)	0.735
TB (µmol/L)	16.10 (12.30, 21.90)	-0.23 (-0.29, -0.16)	< 0.001 ^***^
ALT (U/L)	19.70 (16.70, 23.70)	-0.64 (-0.74, -0.66)	< 0.001 ^***^
AST (U/L)	17.00 (13.50, 23.20)	-0.16 (-0.21, -0.11)	< 0.001 ^***^
ALP (U/L)	68.81 ± 22.26	-0.70 (-0.74, -0.66)	< 0.001 ^***^
FBG (mmol/L)	5.16 ± 1.11	-7.33 (-8.20, -6.46)	< 0.001 ^***^
Serum potassium (mmol/L)	4.19 ± 0.07	-1.22 (-12.35, 9.90)	0.125
Serum calcium (mmol/L)	2.35 ± 0.04	-19.34 (-47.13, 8.46)	0.172
Serum phosphorus (mmol/L)	1.17 ± 0.03	1.49 (-30.65, 33.63)	0.927
GH (%)	5.84 ± 0.66	-14.21 (-15.94, -13.00)	< 0.001 ^***^

DBP, diastolic blood pressure; SBP, systolic blood pressure; BMI, body mass index; TC, total cholesterol; HDL-C, high-density lipoprotein cholesterol; TG, triglycerides; LDL-C, low-density lipoprotein cholesterol; TP, total protein; TB, total bilirubin; ALP, alkaline phosphatase; ALT, alanine aminotransferase; AST, aspartate aminotransferase; GH, glycosylated hemoglobin; FBG, fasting blood glucose; n, number of subjects; %, weighted percentage

^*^P < 0.05, ^**^P < 0.01, ^***^P < 0.001.

### Relationship between TC levels and lumbar BMD

**Fig. 3:**
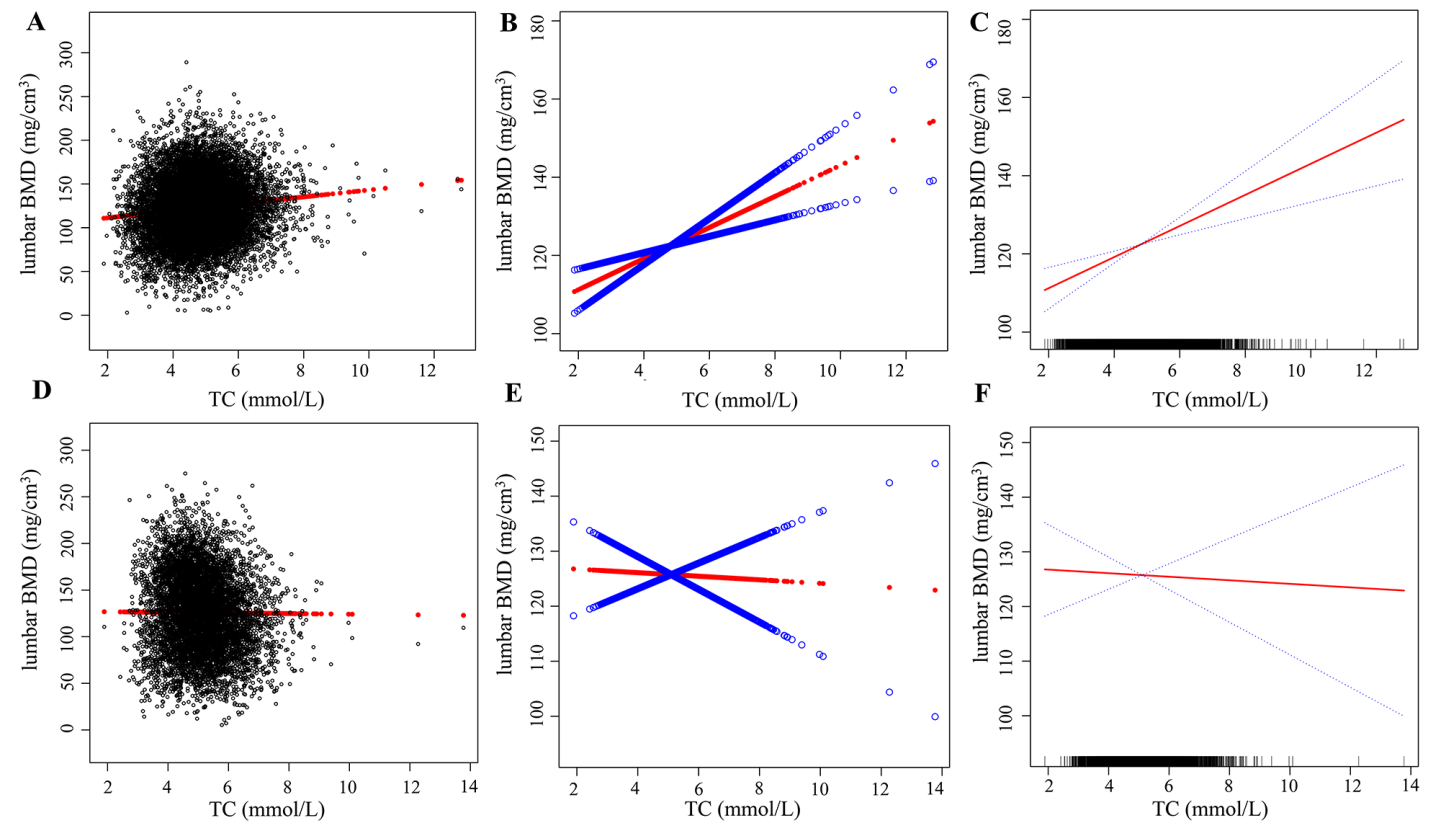
Relationship between TC and lumbar BMD. **A-C** for male, **D-F** for female. **A** and **D**: Each black hollow point exhibits one participant. **B**, **C,E**, and **F**: Solid red line illustrates the fitted smooth curve among variables. Age, nationality, marital status, DBP, SBP, BMI, HDL-C, TG, LDL-C, TP, TB, serum phosphorus, serum calcium, serum potassium, ALP, ALT, AST, GH, and FBG were adjusted

Table 3 presents the findings of the three multiple linear regression models. No confounding factors were adjusted in the current study. Age and nationality were adjusted for in Model 1, and all potential confounding variables were controlled for in Model 2. In this study, there was a positive correlation between serum TC level and lumbar BMD in the current male model (*β* = 3.169, 95% CI: −2.583 to − 3.756, *P* < 0.001) and Model 2 (*β* = 3.978, 95% CI:2.088 to 5.867, *P* < 0.001), whereas Model 1 (*β* = −0.257, 95% CI: −0.752 to − 0.239, *P* = 0.309) showed no significant correlation. Serum TC was negatively correlated with lumbar BMD in current female models (*β* = −5.721, 95% CI: −6.732 to − 4.710, *P* < 0.001). The same relationship persisted in Model 1 after adjusting for covariates (*β* = −1.423, 95% CI: − 2.390 to − 0.961, *P* < 0.001), but no significant association was found in Model 2 (*β* = −0.269, 95% CI: −3.409 to 2.067, *P* = 0.842). Smooth curves of serum TC and lumbar BMD are shown in Fig. 3. Serum TC level was reclassified as a categorical variable with four intervals instead of being treated as a continuous variable. This conversion was performed to examine the correlation between TC and other variables at different concentration ranges. With group Q1 as the control, the regression analysis yielded the analysis results of the three models in the concentration intervals of Q2, Q3, and Q4. Based on these findings, Model 1 exhibited a negative correlation between serum TC and lumbar BMD in the female cohort (Q2: *β* = −2.333, 95% CI: −4.226 to − 0.439, *P* < 0.001; Q3: *β* = −2.787, 95% CI: −4.681 to − 0.893, *P* = 0.004; Q4: *β* = −4.171, 95% CI: −6.072 to − 2.271, *P* < 0.001), and the trend test showed *P* < 0.001. After adjusting for all covariates (Model 2), no significant relationship was found between serum TC level and lumbar BMD in either male or female.

**Table 3:** Relationship between serum TC and lumbar BMD

	Crude model		Model 1		Model 2	
	*β* (95% CI)	*P* value	*β* (95% CI)	*P* value	*β* (95% CI)	*P* value
Male						
TC (mmol/L)	3.169(2.583, 3.756)	<0.001 ^***^	-0.257(-0.752, 0.239)	0.309	3.978(2.088, 5.867)	<0.001 ^***^
Q1	Reference		Reference		Reference	
Q2	6.221 (4.590, 7.852)	<0.001^***^	-0.776(-2.144, 0.591)	0.265	0.551(-1.107, 2.208)	0.538
Q3	6.651(5.024, 8.277)	<0.001^***^	-1.071(-2.437, 0.296)	0.125	0.914 (-1.177, 3.005)	0.401
Q4	8.340(6.713, 9.967)	<0.001^***^	-0.833(-2.205, 0.540)	0.234	2.324(-0.584, 5.232)	0.117
*P* for trend		<0.001 ^***^		0.212		0.145
Female						
TC (mmol/L)	-5.721 (-6.732, -4.710)	<0.001 ^***^	-1.423(-2.390, -0.961)	<0.001 ^***^	-0.269(-3.409, 2.067)	0.842
Q1	Reference		Reference		Reference	
Q2	2.253 (-0.517, 5.024)	0.103	-2.333(-4.226, -0.439)	0.016^*^	-1.292(-3.513, 0.929)	0.242
Q3	-5.589(-8.362, -2.815)	<0.001^***^	-2.787(-4.681, -0.893)	0.004^**^	-1.391(-4.252, 1.470)	0.340
Q4	-14.203(-16.969, -11.437)	<0.001^***^	-4.171(-6.072, -2.271)	<0.001^***^	-2.405(-6.481, 1.672)	0.243
*P* for trend		<0.001 ^***^		<0.001 ^***^		0.305

Crude model: no covariates were adjusted.

Model 1: Age and nationality were adjusted.

Model 2: Age, nationality, marital status, DBP, SBP, BMI, HDL-C, TG, LDL-C, total protein, total bilirubin, serum phosphorus, serum calcium, serum potassium, alkaline phosphatase, ALT, AST, glycosylated hemoglobin, and FBG were adjusted.

^*^P < 0.05, ^**^P < 0.01, ^***^P < 0.001

### Subgroup analysis

In subgroup analyses stratified by age, serum TC level was positively associated with lumbar BMD in men aged < 45 years (*β* = 3.466, 95% CI:0.039–6.893, *P* = 0.047) and ≥ 45 years (*β* = 3.681, 95% CI:1.219–6.144, *P* = 0.0031). In women, serum TC level was negatively associated with lumbar BMD at age ≥ 45 years (*β* = −4.122, 95% CI: −11.706 to 3.462, *P* = 0.005). BMI was transformed into a grouped variable using 24 and 28 as cut-off points. In the male cohort with a BMI between 24 and 28, serum TC was positively correlated with lumbar BMD when stratified by BMI (*β* = 5.270; 95% CI,2.692–7.848; *P* < 0.001), while no significant association was observed in the female group. Interaction analysis showed that BMI and age affected the relationship between serum TC and lumbar BMD in males; however, only age had an effect in the female cohort (Table 4). In the male cohort, the relationship between serum TC and lumbar BMD displayed the greatest strength in individuals aged ≥ 45 years with BMI ranging from 24 to 28 kg/m^2^.

**Table 4:** Serum TC and lumbar BMD were subgroup analyzed and stratified by age and BMI.

Subgroup analysis	*β* (95% CI)	*P* value	*P* for interaction
Male			
Age, year			<0.001 ^***^
Age < 45	3.466 (0.039, 6.893)	0.047^*^	
Age ≥ 45	3.681 (1.219, 6.144)	0.003^**^	
BMI (kg/m^2^)			<0.001 ^***^
< 24	3.525 (-0.709, 7.759)	0.102	
≥ 24,<28	5.270 (2.692, 7.848)	<0.001 ^***^	
≥ 28	2.004 (-1.692, 5.700)	0.288	
Female			
Age, year			<0.001 ^***^
Age < 45	-4.122 (-11.706, 3.462)	0.288	
Age ≥ 45	-5.076 (-8.621, -1.531)	0.005^**^	
BMI (kg/m^2^)			0.160
< 24	-0.709 (-4.499, 3.081)	0.714	
≥ 24,<28	-0.091 (-4.345, 4.164)	0.967	
≥ 28	-1.780 (-9.676, 6.116)	0.658	

Each stratification was adjusted for all factors (age, nationality, marital status, DBP, SBP, BMI, HDL-C, TG, LDL-C, total protein, total bilirubin, serum phosphorus, serum calcium, serum potassium, alkaline phosphatase, ALT, AST, glycosylated hemoglobin, and FBG), except for the stratification factor itself.

*P < 0.05, **P < 0.01, ***P < 0.001

### Non-rectilinear relationship analysis

**Fig. 4:**
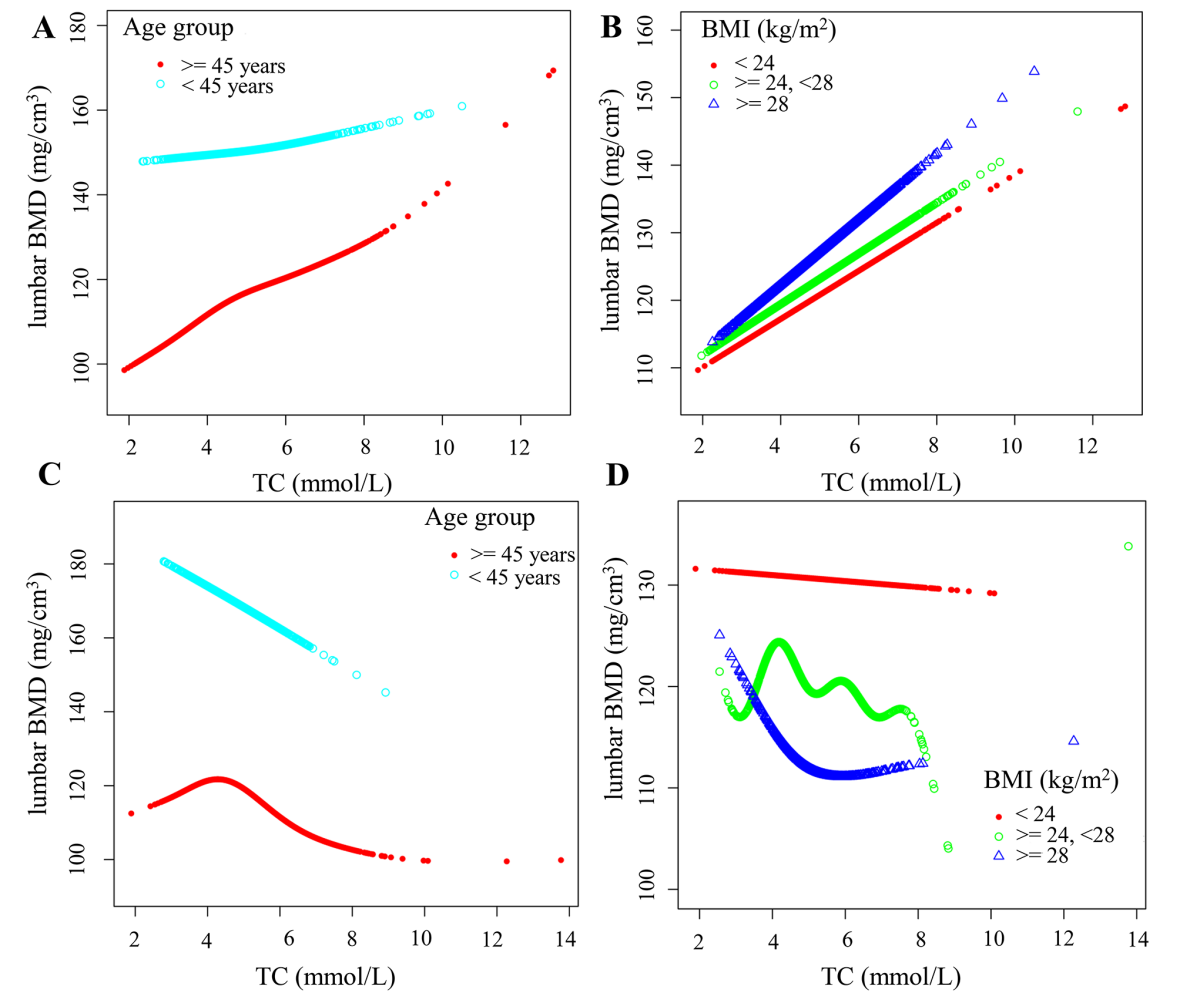
Association between serum TC and lumbar BMD stratified by tertiles of age and BMI. **A-B** for male, **C-D** for female. **A** and **C**: The relationship between TC and lumbar BMD stratified by nationality, marital status, DBP, SBP, BMI, HDL-C, TG, LDL-C, TP, TB, serum phosphorus, serum calcium, serum potassium, ALP, ALT, AST, GH, and FBG. **B** and **D**: The relationship between TC and lumbar BMD stratified by BMI, age, nationality, marital status, DBP, SBP, HDL-C, TG, LDL-C, TP, TB, serum phosphorus, serum calcium, serum potassium, ALP, ALT, AST, GH, and FBG.

In addition, we performed piecewise linear regression and smoothed curve fitting for the age- and BMI-stratified subgroups (Fig. 4; Table 5). Fig. 4C illustrates the point of inflection in the fitted curve for females below the age of 45 years with a serum TC level of 4.27 mmol/L. Fig. 4D shows the inflection point when the BMI of female was greater than 28 and the serum TC was 4.35 mmol/L. Based on the stratified analysis of age and BMI in the male cohort, no non-linear relationship was found between serum TC and lumbar BMD (Fig. 4A and B).

**Table 5:** Multivariate regression analysis of the effect of TC on BMD in different gender populations

	Linear regression	Break point (K)	< K	> K	LLR test
	*β* (95% CI)		*β* (95% CI)	*β* (95% CI)	*P*
Male					
Age					
< 45	3.466 (0.039, 6.893)	5.10	2.276 (-1.896, 6.449)	4.147 (0.460, 7.835)	0.325
≥ 45	3.678 (1.214, 6.142)	4.44	5.649 (2.691, 8.606)	3.113 (0.605, 5.621)	0.180
BMI					
< 24	3.525 (-0.709, 7.759)	5.53	4.098 (-0.363, 8.559)	2.873 (-1.652, 7.399)	0.423
≥ 24, < 28	5.270 (2.692, 7.848)	5.19	4.801 (1.966, 7.636)	5.715 (2.905, 8.524)	0.434
≥ 28	2.004 (-1.692, 5.700)	3.34	11.193 (-2.401, 24.788)	1.842 (-1.860, 5.544)	0.167
Female					
Age					
< 45	-2.517(-9.616, 4.582)	3.64	11.878 (-5.680, 29.436)	-3.036 (-10.153, 4.081)	0.079
≥ 45	-5.076(-8.621, -1.531)	4.27	6.581 (0.874, 12.287)	-5.961 (-9.514, -2.407)	< 0.001^***^
BMI					
< 24	-0.709 (-4.499, 3.081)	6.24	-1.285 (-5.246, 2.675)	0.726 (-4.019, 5.470)	0.323
≥ 24, < 28	-0.091 (-4.345, 4.164)	4.08	7.441 (-0.526, 15.408)	-0.524 (-4.793, 3.745)	0.028^*^
≥ 28	-1.780 (-9.676, 6.116)	4.35	-15.682 (-28.066, -3.298)	-0.382 (-8.292, 7.529)	0.004^**^

Multivariate linear regression model adjusted for age, nationality, marital status, DBP, SBP, BMI, HDL-C, TG, LDL-C, total protein, total bilirubin, serum phosphorus, serum calcium, serum potassium, alkaline phosphatase, ALT, AST, glycosylated hemoglobin, and FBG.

*P < 0.05, **P < 0.01, ***P < 0.001

## Discussion

The goal of this investigation was to examine the correlation between serum TC levels and lumbar BMD in Chinese people. A highly homogeneous sample (n = 20,544) aged 20–80 years, examined by the physical examination department for five consecutive years, was used in this study. After adjusting for age, nationality, and several other variables, this study found a positive correlation between serum TC levels and lumbar BMD in men. In contrast, a negative correlation between serum TC and lumbar BMD was observed in a female cohort aged ≥ 45 years with TC > 4.27 mmol/L, and a positive correlation between serum TC and lumbar BMD was found when BMI ≥ 28 and TC > 4.35 mmol/L. Therefore, the association between serum TC and lumbar BMD differs significantly between the sexes and is affected by both age and BMI.

The relationship between serum TC level and BMD in adults has received considerable attention. A mounting body of biological and epidemiological evidence supports the link between cardiovascular disease and osteoporosis [12], and lipid metabolism has been implicated in the progression of both conditions. Serum TC is a metabolite of cyclopentane dihydrophenanthrene, which plays a significant role in tissue cell metabolism. Epidemiological studies have shown that TC levels in adult plasma are increasing [13]. However, the exact mechanism of interaction between TC and BMD is unclear, and the correlation between the two remains controversial [6, 14]. Bone is extensively innervated and vascularized, appearing to be a self-contained system; however, it is intricately linked to systemic metabolic homeostasis and is subject to dynamic regulation by hormones and nutrients. Bone metabolism, which is mediated by osteoblasts, osteocytes, and osteoclasts, is an ongoing process in bone formation and resorption. Cholesterol and its metabolites influence bone homeostasis by regulating osteoblast and osteoclast differentiation and activation [15]. Studies have indicated that by inhibiting cholesterol biosynthesis, it is possible to inhibit the mRNA expression of bone marrow cells, which serve as precursors to osteoblasts. This can effectively hinder osteogenic differentiation and increase BMD [3, 6]. Elevated total cholesterol levels may result in the buildup of blood vessels within the endothelial matrix of the bone and hinder both osteoblast differentiation and mineralization [16].

In this study, after adjusting for covariates, a positive correlation between serum TC and lumbar BMD was observed in men, with the strongest correlation observed in men aged ≥ 45 years and with a BMI between 24 and 28 kg/m^2^. Another study of data obtained from Chinese adults aged ≥ 65 years showed that BMI was a significant mediator of the positive correlation between blood lipids and lumbar BMD [17]. Obesity is associated with abnormal lipid metabolism [18], and it is generally measured by BMI. In some studies, BMI was independently correlated with BMD [19]. TC is the principal output of fat metabolism. An increase in BMI leads to greater mechanical load, thereby stimulating bone metabolism and increasing BMD. Leptin is a hormone derived from fat cells. In vitro research has illustrated that leptin can directly influence mesenchymal stem cells within the bone marrow, encouraging their development into osteoblasts while preventing their differentiation into adipocytes [20]. A study conducted on obese mice revealed that peripheral administration of leptin resulted in increased bone mass by inhibiting bone resorption and boosting bone formation [21]. In the Spanish Camargo cohort, serum TC showed positive relationship with BMD at the lumbar and hip areas in male individuals aged > 50 years [22], the findings of the investigation corroborate the inferences drawn from this study. Therefore, increased BMI leads to increased leptin secretion and BMD. However, a study utilizing NHANES data acquired from 2011 to 2018 in the United States demonstrated that serum TC was inversely correlated with BMD among men aged 20–59 years [5]. A study of cancer-free older adults in the United States revealed a significant negative correlation between serum TC levels and lumbar BMD in men aged > 60 years [4]. The reason for this difference may be the difference in eating habits and lifestyles between Asians, Europeans, and Americans. Another study from the Framingham cohort in the US found no significant correlation between TC and BMD in man aged 32–61 years even after adjusting for covariates such as age, smoking, alcohol consumption, BMI, SBP, diabetes, and estrogen use [23]. The above conclusions remain controversial, and these studies have shortcomings, such as the lack of homogeneity of the selected population, small sample size, and differences between the adjusted variables. However, this study overcame these limitations.

In the female cohort of this study, a non-linear relationship between serum TC and lumbar BMD was found, and a negative correlation was observed when age ≥ 45 years and serum TC ≥ 4.27 mmol/L. When BMI was ≥ 28 and serum TC was ≥ 4.35 mmol/L, serum TC was positively correlated with lumbar BMD. Age is an important factor affecting BMD changes, and lack of estrogen after menopause is the main factor in women losing bone as they age [24]. Thus, estrogen decline in women aged ≥ 45 years may mediate the relationship between serum TC levels and lumbar BMD. Qi et al. collected 1,116 Chinese women in their 30s and found a non-linear relationship between TC and BMD, with a negative correlation to the left of the inflection point (5.86 mmol/L) and a positive correlation to the right [6]. There were many similarities between this study and the present study, but the results were different in that the present study had more participants, including more biochemicals as covariates than their research. Most importantly, the present study had subgroup analyses of age and BMI of the participants, which was probably the biggest difference between the two studies. The present study identified an inflection point in the age- and BMI-mediated relationship between TC and BMD, which was of inestimable significance and may constitute the true relationship between the two and was expected to lay the foundation for further research into the non-linear relationship between TC and BMD. Another large study in a Chinese population aged 25–64 years found a negative linear regression relationship between BMD and lipids (TC, HDL-C, LDL-C, and TG) after adjusting for several covariates [25], this was different from the results of this study, in which there was a non-linear relationship between TC and BMD. Based on data from 10,402 women who underwent lipid profile (TC, LDL-C, HDL-C, and TG) and BMD measurements at the Korean Health Care System Centre, Jeong et al. found no significant correlation between lipid profiles and BMD after adjusting for potential confounders [26]. However, a study based on NHANES data from 1996 to 2006 in the United States demonstrated a negative correlation between serum TC and lumbar BMD in females, aged 20–85 years, and the strongest negative correlation was primarily found in women aged ≥ 45 years with a BMI < 24.9 kg/m^2^ [27]. In this study, there was a positive correlation between serum TC and lumbar BMD in women with BMI ≥ 28 and TC > 4.35 mmol/L. The abovementioned studies suggest that TC control strategies differ among Chinese women of different ages and BMI. Older women and women with low BMI may require close monitoring of BMD and early intervention.

### Study strengths and limitations

This study has several advantages. First, the samples were obtained from the same region with strong homogeneity and a large sample size; therefore, the research conclusions were more reliable. Second, this study conducted separate statistics for different sexes, making the results more suitable for generalization in the population. In addition, due to the extensive sample sizes, this study evaluated potential sex disparities between serum TC and lumbar BMD while stratifying them based on age and BMI. However, this study had some limitations. First, this study did not collect participants’ exercise and diet information, or other covariates included in the study. Furthermore, establishing a causal link between serum TC levels and lumbar BMD is challenging because this study was cross-sectional. Moreover, the population of this study did not cover the entire population of China because this research selected physical examination samples from one province, and samples from multiple centers still need to be included for result verification. Therefore, longitudinal studies with substantial sample sizes are necessary to investigate the role of serum TC in bone metabolism.

## Conclusion

In a uniform Chinese physical examination cohort, serum TC was found to have a positive correlation with lumbar BMD in men aged 45 ≥ years with BMI between 24 and 28 kg/m^2^ after adjusting for covariates. In the female cohort aged ≥ 45 years, a negative relationship between serum TC level and lumbar BMD was observed when serum TC > 4.27 mmol/L. When BMI ≥ 28 kg/m^2^ and serum TC > 4.35 mmol/L, A positive correlation between serum TC and lumbar BMD was observed when BMI was ≥ 28 kg/m2 and serum TC was > 4. The findings of this study suggest that serum TC and lumbar BMD were differently affected by BMI and age across sex cohorts, and the strategies for controlling blood lipid levels in different populations are different. The combination of BMI and age factors across sexes influenced the process of osteoblast synthesis and differentiation, which in turn affected BMD; however, the exact mechanism of action still needs to be elucidated by further studies. The results of this study provide a reference for BMD monitoring in the Chinese adult population and help clinical nurses identify groups who are at high risk for BMD decline as early as possible and to intervene at an early stage.

### Electronic supplementary material

Below is the link to the electronic supplementary material.

Supplementary Material 1

Supplementary Material 2

## Data Availability

Contact the first author for all data relating to this study.
