# Supplementary material for: Association between total cholesterol and lumbar bone density in Chinese: a study of physical examination data from 2018 to 2023
Source: Lipids Health Dis. 2023 Oct 21;22:180. doi: 10.1186/s12944-023-01946-5 (PMC10590520; doi:10.1186/s12944-023-01946-5)
Supplement: Supplementary file 1 — Supplementary Material 1 [file 12944_2023_1946_MOESM1_ESM.pdf]

Certificate of English Editing

To whom it may concern:

This memo certifies that one of our clients has contracted our academic editing service for the following file.

Title of the paper:
Association between total cholesterol and lumbar bone density in Chinese: a study of physical examination data from 2018 to 2023
Date of the review:
10/09/23 (MM/DD/YY)

The English review was conducted using a two-stage process, in which a junior editor first reviewed the file, and then a senior editor conducted a final and more thorough review. All of our editors are native English-speaking professionals.

Documents receiving this certification should be English-ready for publication; however, the author has the ability to accept or reject our suggestions and changes.

We would like to emphasize that our service targets grammar and language edits. We do not rewrite the documents from scratch. If you are dissatisfied with specific revisions, please contact service@essaystar.com.

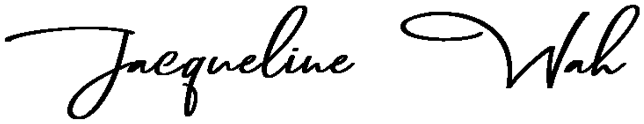

Essaystar Group

+1-208-975-4235
EssayStar, 93 S Jackson St, Seattle, WA 98104
