# Supplementary material for: Association between total cholesterol and lumbar bone density in Chinese: a study of physical examination data from 2018 to 2023
Source: Lipids Health Dis. 2023 Oct 21;22:180. doi: 10.1186/s12944-023-01946-5 (PMC10590520; doi:10.1186/s12944-023-01946-5)
Supplement: Supplementary file 2 — Supplementary Material 2 [file 12944_2023_1946_MOESM2_ESM.pdf]

20231009195100908846096859426816

WORD COUNT 4257
TIME SUBMITTED 09-OCT-2023 07:52PM
PAPER ID 103345274

Association between total cholesterol and lumbar bone density in Chinese: a study of physical examination data from 2018 to 2023

Abstract

Background: The impact of total cholesterol (TC) on lumbar bone mineral density (BMD) has been a topic of interest. However, empirical evidence on this association from demographic data surveys conducted in China is lacking. Therefore, this study aims to examine the relationship between serum TC and lumbar BMD in a sample of 20,544 Chinese adults between the ages of 20 and 80 years, over a period of 5 years, from February 2018 to February 2023. Thus, to investigate the effect of serum TC on lumbar BMD and its relationship with bone reduction in a Chinese adult population.

Methods: This study employed a cross-sectional analysis utilizing data obtained from the Department of Health Management at Henan Provincial People's Hospital between February 2018 and February 2023. The aim of this research was to examine the correlation between serum TC and lumbar BMD in individuals of different genders. The research methodology encompassed population description, an analysis of stratification, single-factor and multiple-equation regression analyses, smooth curve fitting, analysis of threshold and saturation effects. The R and EmpowerStats software packages were used to this statistical analysis.

Results: Multiple linear regression model showed that after adjusting for confounding variables, there was a significant correlation observed between TC and lumbar BMD in men subjects. In subgroup analysis, serum TC was found to have a positive association with lumbar BMD in men, specifically those aged 45 years or older with a body mass index (BMI) ranging from 24 to 28 kg/m^2. A U-shaped correlation arose between serum TC and lumbar BMD was detected in women of different ages and BMI, the inflection point was 4.27mmol/L for women aged ≥ 45 years and 4.35 mmol/L for women with a BMI of ≥ 28 kg/m^2.

Conclusion: In this study, Chinese adults aged 20 to 80 years displayed different effects of serum TC on lumbar BMD in gender-specific populations. Therefore, monitoring BMI and serum TC levels in women of different ages could prevent osteoporosis or osteopenia.

Trial registration: The research protocol has obtained ethical approval from the Ethics Committee at Beijing Jishuitan Hospital in accordance with the Declaration of Helsinki guidelines (No. 2015-12-02). This data is part of the China Health Quantitative CT Big Data Research team, which has been registered in clinicaltrials.gov (code: NCT03699228).

Keywords: osteoporosis, bone mineral density, total cholesterol, Chinese adults

Introduction

Total cholesterol (TC) is a vital lipid constituent in the human body that

has a critical impact on bone cell metabolism [1]. Recent studies have proposed the concept of cholesterol toxicity, which influences organs through activation of inflammation, mitochondrial dysfunction, and endoplasmic reticulum stress [2]. A preclinical study using quantitative computed tomography in mice found that a high-cholesterol diet could lead to low bone content [3]. A research based on the data from the US National Health and Nutrition Examination Survey (NHANES) database have shown that TC is inversely associated with lumbar bone mineral density (BMD) in older non-cancer individuals aged ≥ 60 years [4]. Another study discovered a correlation between low TC levels and reduced total BMD in young adults aged 20-29 years; in individuals aged 40-49 years and those with borderline diabetes, a non-linear curve associating TC with total BMD was identified, with an inflection point at 4.65 mmol/L and 6.7 mmol/L, respectively. [5]. A study of 1,116 Chinese women showed a non-linear association between TC and lumbar BMD among postmenopausal women, and a negative correlation between them when TC < 5.86 mmol/L [6]. All the above-mentioned studies suggest that TC may be related to BMD, but few data have been obtained from China. Therefore, exploring the correlation between serum TC and lumbar BMD in Chinese from a holistic perspective is necessary.

Osteoporosis is a chronic condition marked by a heightened prevalence of generalized bone mineral density (BMD) loss, and around 200 million

people worldwide [7] and approximately 90 million people in China suffer from this disease [8]. Osteoporosis is commonly classified into primary and secondary types, and BMD reduction can be used as a common diagnostic index [9]. Researchers typically evaluate the progression of osteoporosis through lumbar BMD measurements. Given the high prevalence and harm of osteoporosis, investigating how TC affects BMD is critical. Studies indicated that women are generally at greater risk of osteoporosis than men, and that most men tend to have larger, stronger bones than women and experience less bone loss throughout their lives [10]. In recent years, the relationship between TC and BMD has been explored and concerned worldwide, but the results remain inconsistent because of geographical and sample size limitations, which indicates that race has a great impact on the prevalence of osteoporosis. Therefore, the homogenous study population and reasonable sample size in this study can assist in clarifying the effect of TC on BMD in Chinese adults. This information may offer guidance in preventing and treating BMD loss in the Chinese adults. To our knowledge, this research represents the first investigation of the association between lumbar BMD and TC in a Chinese population of different sexes, using a large dataset of medical examinations and performing a subgroup analysis.

This study gathered data from individuals who received physical examinations over 5 consecutive years at Henan Provincial People's

Hospital in China to explore the linear or non-linear correlation between TC and BMD in varying genders. Covariates such as blood pressure and BMD-related biochemical test results were filtered to improve sample quality and corresponding analysis.

Materials and methods

Study participants and inclusion criteria

The study's analysis relied on the physical examination data from the Health Management Department of Henan Provincial People's Hospital, collected between February 2018 and February 2023. This data is part of the China Health Quantitative CT Big Data Research project team, which has been registered in clinicaltrials.gov (code: NCT03699228). Inclusion criteria were: (1) age between 20–80 years; (2) complete information of lumbar BMD and blood biochemical examination; (3) complete body mass index (BMI) and general demographic information. The following criteria were used to exclude: (1) history of various cancers; (2) previous or current thyroid disease and other endocrine diseases; (3) previous or current liver or kidney disease; (4) past or present use of osteoporosis-preventing drugs and lipid-regulating agents. The trained personnel obtained fundamental data through in-person surveys, including age, gender, nationality, medical history, and medication history of the patients.

A total of 23,653 participants were collected, 123 of whom were aged < 20 years and, thus, were excluded from the study. In addition, 2569 patients

had incomplete lumbar BMD or serum TC or BMI, and 417 had medical histories that did not meet the inclusion criteria. Finally, the study included 20,544 participants. The subject screening flow chart is displayed in **Fig. 1**.

Research Methods

All researchers were given uniform training before the investigation to ensure the precision and impartiality of the data. A standardized questionnaire was utilized for gathering fundamental data about the participants. including patient's prior and present medical history, such as a history of cancer, liver disease, kidney disease, thyroid, and other endocrine disorders, and the use of anti-osteoporosis drugs and any lipid metabolism regulators. The data were summarized, checked, verified, and proofread after the completion of the questionnaire.

Participants' height, weight, and blood pressure were measured in the morning after fasting for over 12 hours with light clothing and no shoes. Each subject was measured two times and then averaged to reduce error. BMI= weight divided by height^2 (kg/m^2).

Laboratory Measurements

Fasting blood samples were collected to measure TC, as well as other laboratory markers, including high-density lipoprotein cholesterol (HDL-C), triglycerides (TG), low-density lipoprotein cholesterol (LDL-C), total protein (TP), total bilirubin (TB), blood phosphorus, blood potassium, blood calcium, alkaline phosphatase (ALP), alanine aminotransferase

(ALT), aspartate aminotransferase (AST), fasting blood glucose (FBG), and glycated hemoglobin (GH). An Olympus® AU 5400 automated biochemical analyzer (Olympus Corporation, Japan, Shizuoka) was used to assess blood glucose and lipids. Conventional laboratory techniques were used to assess the remaining indicators.

BMD Measurement

Low-dose chest CT (LDCT) scanning formed part of the participants' routine health examination, with each participant undergoing the same LDCT procedure. Quantitative Computed Tomography (QCT) vBMD was measured using Mindways QCT Pro (Mindways Software, Inc., Austin, USA), while all CT scans were performed at 120 kVp. LDCT images were sent to the QCT station for analysis. Lumbar (L1-L2) trabecular vBMD (mg/cm^3) was determined using asynchronous BMD calibration and QCT Pro analysis (Mindways Software, Inc., Austin, USA). All analyses were performed by QCT software experienced and trained radiologists. This procedure necessitates the post-imaging analysis of conventional LDCT images. Therefore, no extra radiation dose is incurred. A study, which has already been published, validates that these criteria are appropriate for the Chinese people [11].

Quality control was upheld consistently during the research period via routine calibration and cross-calibration between systems, utilizing a European spinal prosthesis (ESP-145). The results of quality assurance

indicated that the mean variation in ESP vBMD detected at each center was under 5 mg/cm^3.

Variables

In this investigation, TC and BMD were employed as the independent and dependent variable, correspondingly. The following categorical variables were included as covariates: nationality and marital status. The following dimensions were included as constant covariates in this analysis: age, BMI, systolic blood pressure (SBP), diastolic blood pressure (DBP), HDL-C, TG, LDL-C, TP, TB, blood phosphorus, blood potassium, blood calcium, ALP, ALT, AST, FBG, and GH.

Statistical analysis

All data were analyzed utilizing EmpowerStats (X&Y solutions, Inc., Boston, MA) and the statistical packages R (The R Foundation; version 3.6.3). In the final evaluation, baseline characteristics of all subjects were described by means or medians and quartiles (continuous variable) or proportions (categorical variable). The chi-squared test and variance estimation were employed to deal with significant discrepancies in this dataset. The connection between serum TC and lumbar BMD was examined through a multiple linear regression model. The study employed a multivariate linear regression model to perform subgroup analysis of the linear relationship between serum TC and lumbar BMD among diverse gender groups, categorized by BMI and age. The smooth curve fitting and

the generalized additivity model were used to define the nonlinear correlation between serum TC and lumbar BMD. For situations of nonlinearity in the model, objective calculations were conducted to determine the point of inflection in the correlation between serum TC and lumbar BMD, using a recursive algorithm, the two-stage linear regression model was then established on either side of this point. Statistical significance was achieved when the two-tailed $P < 0.05$.

The serum TC frequency distribution graph was created with Origin software (OriginLab, USA, version: 2022b).

Result

Participant baseline characteristics

A total of 20,544 individuals aged 20 to 80, comprising 13,389 males and 7,155 females, were recruited for the research. The characteristics of male and female participants were determined by serum TC (Q1: 1.88–4.14, Q2: 4.15–4.77, Q3: 4.78–5.41, Q4: 5.42–12.83; Q1: 1.89–4.44, Q2: 4.45–5.04, Q3: 5.05–5.7, Q4: 5.71–13.77) divided into quartiles. As presented in **Table 1**, notable variations in baseline characteristics were noted between quartiles of serum TC, aside from nationality, serum phosphorus, and serum potassium in men, and nationality and serum potassium in women. Compared with the other subgroups, it's likely that the men with the highest fourth of serum TC levels were younger, Han, and married, with higher SBP, DBP, lipid indices (HDL-C, TG, LDL-C, and

TP), ALP, ALT, AST, FBG, and lumbar BMD; participants with the highest quartile of serum TC in women were probably older, Han, and married, with higher SBP, DBP, lipid indexes (HDL-C, TG, LDL-C, and TP), TP, serum calcium, serum potassium, ALP, ALT, AST, GH, FBG, and lumbar BMD. The serum TC distribution of all participants, males, and females, is shown in **Fig. 2**.

Univariate analysis

Univariate analysis demonstrated a negative correlation between lumbar BMD and age, marital status, SBP, HDL-C, ALP, FBG, serum potassium, and GH in the male cohort. On the contrary, BMI, DBP, LDL-C, TG, TP, ALT, and AST demonstrated a favorable correlation with elevated lumbar BMD. In the female cohort, age, marital status, BMI, DBP, SBP, LDL-C, TG, TB, ALT, AST, ALP, FBG, and GH were negatively correlated with lumbar BMD. HDL-C was associated with high lumbar BMD. The results are presented in **Table 2**.

Relationship between TC levels and lumbar BMD

Table 3 presents findings from three multiple linear regression models. No confounding factors were adjusted for in the current model. Age and nationality were adjusted in Model 1, and all potential confounding variables were controlled for in Model 2. In this research, there is a positive correlation between serum TC and lumbar BMD in the current male model (β = 3.169, 95% CI: −2.583 to −3.756, P < 0.001) and Model 2 (β = 3.978,

95% CI: 2.088 to 5.867, $P < 0.001$), whereas Model 1 ($\beta = -0.257$, 95% CI: −0.752 to −0.239, $P = 0.309$) showed no significant correlation. Serum TC exhibited a negative correlation with lumbar BMD in the female current models ($\beta = -5.721$, 95% CI: −6.732 to −4.710, $P < 0.001$). The same relationship persisted in Model 1 after adjusting for covariates ($\beta = -1.423$, 95% CI: − 2.390 to − 0.961, $P < 0.001$), but no significant association was found in model 2 ($\beta = -0.269$, 95% CI: −3.409 to 2.067, $P = 0.842$). The smooth curve of serum TC and lumbar BMD is shown in **Fig. 3**. Serum TC was reclassified as a categorical variable with four intervals, instead of being treated as a continuous variable. This conversion was made to examine the correlation between TC and another variable at differing concentration ranges. With group Q1 as the control, regression analysis yielded the analysis results of the three models in the concentration intervals of Q2, Q3, and Q4. Based on the findings, Model 1 exhibited a negative correlation between serum TC and lumbar BMD among the female cohort (Q2: $\beta = -2.333$, 95% CI: −4.226 to −0.439, $P < 0.001$; Q3: $\beta = -2.787$, 95% CI: −4.681 to −0.893, $P = 0.004$, Q4: $\beta = -4.171$, 95% CI: −6.072 to −2.271, $P < 0.001$), and the trend test showed $P < 0.001$. After adjusting for all covariates (Model 2), no significant relationship was found between serum TC and lumbar BMD in either male or female.

Subgroup analysis

In subgroup analyses stratified by age, serum TC in men was positively

associated with lumbar BMD at age < 45 years ($\beta = 3.466$, 95% CI: 0.039 to 6.893, $P = 0.047$) and ≥ 45 years ($\beta = 3.681$, 95% CI: 1.219 to 6.144, $P = 0.0031$). In women, serum TC was negatively associated with lumbar BMD at age ≥ 45 years ($\beta = -4.122$, 95% CI: −11.706 to 3.462, $P = 0.005$). BMI was transformed into a grouped variable utilizing 24 and 28 as cut-off points. In the male cohort with a BMI between 24 and 28, serum TC was positively correlated with lumbar BMD when stratified by BMI ($\beta = 5.270$, 95% CI: 2.692 to 7.848, $P < 0.001$), while no significant association was observed in the female group. Interaction analysis showed that BMI and age impact the relationship between serum TC and lumbar BMD in males, however, only age has an effect in the female cohort (**Table 4**). In the male cohort, the relationship between serum TC and lumbar BMD displayed the greatest strength in individuals aged ≥ 45 years with a BMI ranging from 24 to 28 kg/m^2.

Non-Rectilinear Relationship Analysis

In addition, this research performed piecewise linear regression and smoothed curve fitting for age- and BMI-stratified subgroups (**Fig. 4** and **Table 5**). **Fig. 4C** illustrates the point of inflection in the fitted curve for females below the age of 45 with a serum TC level of 4.27 mmol/L. **Fig. 4D** shows the inflection point when the BMI of female is greater than 28 and the serum TC is 4.35 mmol/L. Based on the stratified analysis of age and BMI in the male cohort, no non-linear relationship was found between

serum TC and lumbar BMD (**Fig. 4A** and **Fig. 4B**).

Discussion

The goals of this investigation was to examine the correlation between serum TC and lumbar BMD among Chinese people. A highly homogeneous sample (n = 20,544) aged 20–80 years examined by the physical examination department for 5 consecutive years was used in this study. After adjusting for age, nationality, and several other variables, this research found a positive correlation between serum TC and lumbar BMD in men. By contrast, a negative correlation between serum TC and lumbar BMD was observed in the female cohort aged ≥ 45 years with TC > 4.27 mmol/L, and a positive correlation between serum TC and lumbar BMD was found when BMI ≥ 28 and TC > 4.35 mmol/L. Therefore, the association between serum TC and lumbar BMD differs significantly between the genders, and it is affected by both age and BMI.

At present, the relationship between serum TC and BMD in adults has received considerable attention. A mounting body of biological and epidemiological evidence backs the link between cardiovascular disease and osteoporosis [12], and lipid metabolism is implicated in the progression of both conditions. Serum TC is a metabolite of cyclopentane dihydrophenanthrene, which has a significant function in the tissue cells metabolism. Epidemiological studies have shown that TC in adult plasma

is increasing [13]. However, the exact interaction mechanism of TC and BMD is unclear, and the correlation between the two remains controversial [6, 14]. Bone is extensively innervated and vascularized, appearing to be a self-contained system, but it is intricately linked to systemic metabolic homeostasis and subject to dynamic regulation by hormones and nutrients. Bone metabolism is an ongoing process of bone formation and resorption, which is directed by osteoblasts, osteocytes, and osteoclasts. Cholesterol and its metabolites influence bone homeostasis through regulation of osteoblast and osteoclast differentiation and activation [15]. Studies have indicated that by inhibiting cholesterol biosynthesis, it is possible to inhibit the mRNA expression of bone marrow cells, which serve as precursors to osteoblasts. This can effectively hinder osteogenic differentiation and increase BMD [3, 6]. Elevated total cholesterol levels may result in the buildup of blood vessels within the endothelial matrix of bone and hinder both osteoblast differentiation and mineralization [16].

5

In this study, after adjusting for covariates, a positive correlation between serum TC and lumbar BMD was observed in men, with the strongest correlation observed aged 45 years or older and with a BMI between 24 and 28 kg/m^2. Another study of data obtained from Chinese adults aged ≥ 65 showed that BMI was a significant mediator of the positive correlation between blood lipids and lumbar BMD [17]. Obesity is associated with abnormal lipid metabolism [18], and it is generally

measured using BMI. In some studies, BMI is independently correlated with BMD [19]. TC is the principal output of fat metabolism. The increase of BMI will lead to greater mechanical load, thereby stimulating bone metabolism and increasing BMD. Leptin is a hormone derived from fat cells. In vitro research has illustrated that leptin has the capacity to influence mesenchymal stem cells within the bone marrow directly, encouraging their development into osteoblasts while preventing their differentiation into adipocytes [20]. A study conducted on obese mice revealed that the administration of leptin peripherally resulted in increased bone mass by inhibiting bone resorption and boosting bone formation [21]. In the Spanish Camargo cohort, serum TC showed positive relationship with BMD at the lumbar and hip areas in male individuals aged > 50 years [22], the findings of the investigation corroborate the inferences drawn from this study. Therefore, increased BMI leads to the increased secretion of leptin and increased BMD. However, a study utilizing NHANES data acquired from 2011 to 2018 in the United States demonstrated that serum TC had an inverse correlation with BMD amongst men aged 20-59 years [5]. A study of cancer-free older adults in the United States revealed a significantly negative correlation between serum TC levels and lumbar BMD in men with the age > 60 years [4]. The reason for this difference may be the difference in eating habits as well as lifestyles between Asians and Europeans and Americans. Another study from the Framingham cohort

in the US discovered no significant correlation between TC and BMD in man aged 32-61 years even after adjusting for covariates such as age, smoking, alcohol consumption, BMI, SBP, diabetes, and estrogen use [23]. The above conclusions remain controversial, and these studies have shortcomings, such as lack of homogeneity of the selected population, small sample size and differences between the adjusted variables. However, this study overcomes these limitations.

In the female cohort of this study, a non-linear relationship between serum TC and lumbar BMD was found, and a negative correlation was observed when the age ≥ 45 and serum TC ≥ 4.27 mmol/L. When BMI ≥ 28 and serum TC ≥ 4.35 mmol/L, serum TC was positively correlated with lumbar BMD. Age is an important factor affecting BMD changes, and lack of estrogen after menopause is the main factor in women losing bone as they age [24]. Thus, estrogen decline in women age ≥ 45 years may mediate the relationship between serum TC and lumbar BMD. Qi et al. collected 1,116 Chinese women in their 30s and found a non-linear relationship between TC and BMD, with a negative correlation to the left of the inflection point (5.86 mmol/L) and a positive correlation to the right [6]. There were many similarities between this research and the present study, but the results were different in that the present study had more participants, included more biochemicals as covariates than their research, and most importantly the present study had subgroup analyses of age and BMI of the

participants, which was probably the biggest difference between the two studies. The present study identified an inflection point in the age and BMI-mediated relationship between TC and BMD, which was of inestimable significance and may constitute the true relationship between the two and was expected to lay the foundation for further research into the nonlinear relationship between TC and BMD. Another one large study in a Chinese population aged 25–64 years found a negative linear regression relationship between BMD and lipids (TC, HDL-C, LDL-C, and TG) after adjusting for several covariates [25], this was different from the results of this study where there was a non-linear relationship between TC and BMD. Based on data from 10,402 women who underwent lipid profiles (TC, LDL-C, HDL-C, and TG) and BMD measurements at the Korean Health Care System Centre, Jeong et al. found no significant correlation between lipid profiles and BMD after adjusting for potential confounders [26]. However, a study, based on NHANES data from 1996 to 2006 in the United States, demonstrated a negative correlation between serum TC and lumbar BMD in females, aged 20 to 85 years, and the strongest negative correlation was primarily found in women aged ≥ 45 years with BMI < 24.9 kg/m^2 [27]. In this research, there was a positive correlation between serum TC and lumbar BMD in women with BMI ≥ 28 and TC > 4.35 mmol/L. The above-mentioned studies suggested that TC control strategies are different among different age and BMI groups in Chinese women.

Older women and women with a low BMI may require close monitoring of BMD and early intervention.

Study strengths and limitations

This research has some advantages. First, the samples were obtained from the same region with strong homogeneity and a large sample size, so the research conclusion was more reliable. Second, this study conducted separate statistics for different genders, making the results more suitable for generalization in the population. In addition, due to the extensive sample sizes, this study has evaluated potential sex disparities between serum TC and lumbar BMD while stratifying based on age and BMI. However, this study also has some shortcomings. First, this study did not collect participants' exercise and diet information and other included covariables for study. Furthermore, establishing a causal link between serum TC and lumbar BMD is challenging since this study was cross-sectional. Moreover, the population of this study did not cover the whole population of China because this research selected physical examination samples from one province, and samples from multiple centers still need to be included for result verification. Therefore, longitudinal studies with substantial samples are necessary to investigate the role of serum TC in bone metabolism.

Conclusion

In a uniform Chinese physical examination cohort, serum TC was found

to have a positive correlation with lumbar BMD in men aged 45 ≥ years with a BMI between 24 and 28 kg/m^2 after adjusting for covariates. In the female cohort aged ≥ 45, a negative relationship between serum TC and lumbar BMD was observed when serum TC > 4.27 mmol/L. When BMI ≥ 28 kg/m^2 and serum TC > 4.35 mmol/L, a positive correlation between serum TC and lumbar BMD was observed. This study findings suggest that serum TC and lumbar BMD were differently affected by BMI and age across gender cohorts, and the strategies for controlling blood lipid in different populations are different. The combination of BMI and age factors across genders influenced the process of osteoblast synthesis and differentiation, which in turn affected BMD, but the exact mechanism of action still needs to be elucidated by further studies. The results of this study provided a reference for BMD monitoring in Chinese adult population and helped clinical nurses to identify groups who are at high risk for BMD decline as early as possible and to intervene at an early stage.

20231009195100908846096859426816

ORIGINALITY REPORT

9%

SIMILARITY INDEX

PRIMARY SOURCES

1	www.frontiersin.org<br>Internet	129 words — 3%
2	Han Zhang, Kun Ma, Run-Min Li, Jia-ni Li, Shan-feng Gao, Lin-na Ma. "Association between testosterone and bone mineral density in females aged 40-60 years from NHANES 2011- 2016", Research Square Platform LLC, 2022<br>Crossref Posted Content	42 words — 1%
3	Yuanyuan Chen, Jing Xu. "Arm circumference is positively correlated with total femur bone mineral density among adults aged 50 years old and above: The NHANES 2013-2020", Research Square Platform LLC, 2023<br>Crossref Posted Content	34 words — 1%
4	pubmed.ncbi.nlm.nih.gov<br>Internet	30 words — 1%
5	In-Kyong Jeong, Sun Wook Cho, Sang Wan Kim, Hyung Jin Choi et al. "Lipid Profiles and Bone Mineral Density in Pre- and Postmenopausal Women in Korea", Calcified Tissue International, 2010<br>Crossref	28 words — 1%
6	www.science.gov<br>Internet	27 words — 1%
7	bmccardiovascdisord.biomedcentral.com<br>Internet	

25 words — 1%

8 downloads.hindawi.com
Internet
18 words — < 1%

9 www.medrxiv.org
Internet
16 words — < 1%

10 Wang Yu, Wang Jinghong, Deng Pan, Li Jinying, Wang Yiting. "Research on the Fusion Model of Sports and Medicine Based on Artificial Neural Network Health Analysis and Forecast — —Take atherosclerosis as an example", Journal of Physics: Conference Series, 2021
Crossref
14 words — < 1%

11 link.springer.com
Internet
14 words — < 1%

12 bmcendocrdisord.biomedcentral.com
Internet
12 words — < 1%

13 Zhixiong Li, Yongchun Wang, Rui Xing, Huilan Zeng, Xing-Juan Yu, Yao-jun Zhang, Jing Xu, Limin Zheng. "Cholesterol Efflux Drives the Generation of Immunosuppressive Macrophages to Promote the Progression of Human Hepatocellular Carcinoma", Cancer Immunology Research, 2023
Crossref
10 words — < 1%

14 journals.plos.org
Internet
9 words — < 1%

15 Tongsen Ren, Peng Wu, Songjiang Yin, Hankun You, Tan Liu, Zhenghui Li, Qun Liu, Jun Mao. "The association between cardiac metabolic index and lumbar bone
8 words — < 1%

mineral density in adults: NHANES 2011-2018", Research Square Platform LLC, 2023
Crossref Posted Content

EXCLUDE QUOTES	OFF	EXCLUDE SOURCES	OFF
EXCLUDE BIBLIOGRAPHY	ON	EXCLUDE MATCHES	OFF
